# Physical Therapists as Partners for Community Fall Risk Screenings and Referrals to Community Programs

**DOI:** 10.3389/fpubh.2021.672366

**Published:** 2021-06-25

**Authors:** Jennifer L. Vincenzo, Colleen Hergott, Lori Schrodt, Subashan Perera, Jennifer Tripken, Tiffany E. Shubert, Jennifer S. Brach

**Affiliations:** ^1^Department of Physical Therapy, University of Arkansas for Medical Sciences, Fayetteville, AR, United States; ^2^Department of Physical Therapy, Augusta University, Augusta, GA, United States; ^3^Department of Physical Therapy, Western Carolina University, Cullowhee, NC, United States; ^4^Department of Physical Therapy, University of Pittsburg, Pittsburg, PA, United States; ^5^Center for Healthy Aging, National Council on Aging, Washington, DC, United States; ^6^University of North Carolina Center for Health Promotion and Disease Prevention, University of North Carolina, Chapel Hill, NC, United States

**Keywords:** fall prevention, accidental injury, older adult, evidence-based practice, clinical-community connections, partnerships

## Abstract

**Objectives:** Physical therapists (PTs) are integral team members in fall prevention in clinical settings; however, few studies have investigated PTs' engagement in pro-bono community-based falls prevention. Therefore, we aimed to describe the characteristics of PTs and physical therapist assistants (PTAs) in the United States who conduct community-based fall screenings, the reach of screenings, their knowledge and utilization of the Centers for Disease Control and Prevention's fall-risk screening toolkit (STEADI, Stopping Elderly Accidents, Deaths, and Injuries), and therapists' knowledge and referrals to evidence-based programs (EBPs) and community resources.

**Methods:** A cross-sectional survey distributed to a convenience sample of PTs/PTAs in the United States through news-blasts, and social media.

**Results:** Four hundred and forty-four therapists who worked with older adults completed the survey. Approximately 40% of the respondents (*n* = 180) conduct screenings, most frequently annually. People who screen tend to be PTs with >20 years of experience, work in outpatient/wellness or academia, and be involved in the least amount of direct patient care. The majority (*n* = 344, 77.5%) of survey respondents were somewhat to very familiar with the STEADI, and ~84% (*n* = 114) of respondents who were very familiar with the STEADI (*n* = 136) use the toolkit to conduct community-based, pro-bono fall risk screenings. Twenty-six percent (*n* = 14) out of the 53 PTAs who responded to the survey conduct falls screenings in the community. Of the PTs/PTAs who conduct community-based fall screenings (*n* = 180), ~ 75% (*n* = 136) are aware of and refer older adults to EBPs. Over half also refer to Silver Sneakers and/or senior centers.

**Discussion:** PTs and PTAs are key partners in evidence-based multifactorial fall prevention in the community. Data helps inform community organizations that most PTs who engage in community-based fall risk screening utilize the STEADI toolkit and refer to community-based programs. Community organizations seeking PT partners to engage in fall risk screenings and promote referrals to local resources or EBPs will likely have the most success collaborating with local physical therapy education programs or physical therapy clinic managers.

## Introduction

Falls are the leading cause of fatal and non-fatal injuries among older adults in the United States. Approximately 29% of older adults experience a fall annually (1). Between 2007 and 2016 fall-related mortality increased by 31% nationally (2). Annually, the estimated 29 million falls result in seven million injuries and cost the US $50 billion (3). Additionally, older adults who suffer a fall-related injury are more likely to lose their ability to live independently and to be admitted to nursing homes (4). Expanding evidence indicates many falls are preventable and that interventions are cost-effective (5–8). Therefore, it is important to identify avenues to support widespread evidence-based falls prevention.

Evidence-based multifactorial falls prevention and public health toolkits have been developed to facilitate screening and referral of older adults at an increased risk for falls to appropriate intervention programs. The American Geriatrics Society and British Geriatrics Society 2010 clinical practice guideline suggests annual fall risk screenings for all adults 65 years of age and older (9). To facilitate implementation of evidence-based fall risk screenings, assessment, and intervention the Centers for Disease Control and Prevention (CDC) developed the Stopping Elderly Accidents, Deaths, and Injuries toolkit (STEADI) of procedures and materials (10). The STEADI was originally developed for implementation into primary care. However, barriers to implementation resulted in poor uptake. Prior work highlights the complexities of managing medical priorities and workflow issues that limit consistent integration of screenings into primary care practice (11, 12). Additionally, less than half of older adults report falls during primary care visits, and even fewer report discussing fall prevention with their healthcare provider (13). Since falls screenings are not regularly being conducted in primary care, implementation of screenings by other, qualified healthcare providers, such as physical therapy (PTs) and physical therapist assistants (PTAs) in other settings, such as the community, are imperative to address the public health issue of falls among older adults.

Although STEADI was originally developed for integration into primary care, it can be easily implemented in community settings, thereby expanding the reach and access to older adults (14–16). Research supports that PT-led, pro-bono, community fall risk screenings in the United States (US) are not only feasible, but result in older adults' adoption of fall risk reduction behaviors (14, 15, 17). Two separate studies conducted in the US found that older adults who attended a pro-bono, PT-led community fall risk screening using the STEADI toolkit implemented risk reduction strategies following screening and education (14, 15). Conducting fall risk screenings in community settings where older adults commonly congregate may also facilitate participation in evidence-based health promotion/disease prevention programs (EBPs) (6). EBPs are rigorously studied, standardized programs led by trained facilitators that address fall risks such as fear of falling, decreased strength, or decreased balance. EBPs are monitored to maintain program fidelity (18). In recent years, the Administration for Community Living has invested over $14 million to expand and support implementation of EBPs in communities across the country (19). Evidence-based falls prevention programs, such as a Matter of Balance, Stepping On, Tai Chi for Better Balance, and Otago are effective for decreasing multiple risk factors for falls, decreasing falls and fall-related injuries, and are low-cost or free for older adults (6, 20). The National Council on Aging recommends fall prevention EBPs based on falls risk levels and other considerations for the target population (21). Referral to EBPs and other community fall prevention resources is a critical step following screening and a key intervention recommended by STEADI. Community organizations supporting older adults would benefit from building relationships with other healthcare providers who conduct community fall screenings, such as PTs, to increase screenings and awareness of and referrals to EBPs and locally available resources.

Physical therapists and PTAs are key members of the fall prevention team as experts who assess and treat balance, strength, and mobility deficits. Referral to a PT is one of the evidence-based interventions recommended by the CDC and the United States Preventive Services Task Force to manage falls risk (8, 11). Clinical best practice recommendations state that physical therapy providers should routinely screen older adults for fall risk (22). Physical therapists also can help link older adults with community-based programs and resources to increase physical activity, reduce fall risk, or manage chronic diseases (14, 15, 23). Community fall risk screenings by PTs and PTAs, particularly those using the STEADI toolkit, could expand the reach of fall risk screenings and older adults' engagement in community interventions and resources. Although some research is available regarding the impact of PTs conducting pro-bono community fall risk screenings in the US, the reach of these screenings, PTs/PTAs' knowledge and use of the STEADI, and PTs/PTAs' referral of older adults to support falls prevention after screenings are unknown. Identifying these knowledge gaps will increase other healthcare providers and public health professionals' awareness of the role of the PT/PTA in community-based falls prevention in the United States.

This study aims to describe the characteristics of PTs and PTAs in the US who conduct community-based fall risk screenings, the number and geographic reach of screenings, and PTs/PTAs knowledge and utilization of the STEADI toolkit and referrals to EBPs and other community resources.

## Methods

This study was a cross-sectional survey conducted by a task force of experts. The task force developed, pilot tested, and refined the web-based survey to meet the proposed objectives. The data in this paper focuses solely on community fall-risk screenings among PTs and PTAs. The study was deemed exempt by the Institutional Review Board. Ethical approval for this study and written informed consent from the participants of the study were not required in accordance with local legislation and national guidelines. Since the study was exempt, informed consent was not obtained; however, information indicating that the survey was voluntary was included in the introduction and no personal identifying information was obtained.

The survey consisted of 36 questions and took ~20 min to complete. Topics of the questions included (a) demographics such as age, years of experience, practice setting, clinical specializations, and highest degree obtained (16 questions), (b) frequency and number of community fall risk screenings conducted (5 questions), (c) knowledge and use of the CDC's STEADI toolkit (9 questions), and (d) knowledge of and referral to EBPs and other community resources (6 questions). At the time of the survey, the multi-tier (low, medium, high falls risk) screening algorithm was promoted by the CDC (Supplementary Material). The cross-sectional survey was disseminated in the fall of 2019 using Redcap (Research Electronic Data Capture) hosted by the university (24, 25) to a convenience sample of PTs through various methods including email, news-blasts, social media, and sharing by other survey respondents. Emails and news-blasts were explicitly sent through the American Physical Therapy Association (APTA) networks to specific professional specialty areas. There are 18 specialty areas of practice APTA members can join that provide focused resources and networking. APTA members can opt to join multiple specialty areas of practice. The survey was sent through e-blasts to the geriatrics and orthopedics specialty areas of practice and Balance and Falls special interest groups within the specialty practice areas of Geriatrics, Oncology, and Neurology. Physical therapy providers who were not APTA members received the survey through either open social media posts or from a forwarded e-blast.

### Data Analysis

We used appropriate descriptive statistics (means, standard deviations, frequencies, and percentages) to summarize demographic characteristics of the survey respondents. Some categorical variable classes were combined to provide more meaningful classifications or due to small frequencies. To compare characteristics between survey respondents that do and do not conduct fall risk screenings in the community, we used independent samples *t*-tests for continuous data and chi-square and Fisher's Exact tests for categorical data. Additionally, frequency counts and percentages were used to summarize survey responses related to community fall risk screening, and knowledge/use of the STEADI and community interventions. SAS® version 9.4 (SAS Institute, Inc., Cary, North Carolina) was used for all statistical analyses.

## Results

Four hundred and sixty PTs and PTAs participated in the survey between September and November 2019. Incomplete survey responses and PTs who responded but indicated they did not treat older adults were removed from the analysis, resulting in data from 444 PT providers who worked with older adults (88% PTs, 12% PTAs), representing 49/50 states. Table 1 depicts the characteristics of the survey respondents as well as characteristics of members of the APTA. The majority of survey respondents (*n* = 332, 74.8%) were members of the APTA. This is greater than the national percentage, in that ~22% of all physical therapy providers are APTA members (26). Survey respondents' average age was 47.4 years. They were predominantly female (*n* = 366, 82.4%) with a clinical doctorate degree (DPT, *n* = 207, 46.6%). The majority of respondents indicated they were employed full time (*n* = 364, 82.0%), had over 20 years of experience in physical therapy (*n* = 223, 50.2%), and practiced in an outpatient/wellness clinical setting (*n* = 205, 46.2%). Survey participants also indicated the majority of their caseload (*n* = 268, 60.4%) was composed of older adults over the age of 65 years.

**Table 1:** Demographics of survey respondents and American Physical Therapy Association (APTA) members: mean ± standard deviation or *n* (%).

	**Survey respondents *n* = 444**	APTA members 2019^*^n = 73,139
Age	47.4 ± 11.8	42.7
• **Gender** Male Female Prefer not to respond	73 (16.4) 366 (82.4) 5 (1.1)	*n* = 67972 23,517 (34.60) 44,455 (65.40) –
**Occupation**		
Physical therapist Physical therapist assistant	391 (88.1) 53 (11.9)	65,469 (89.51) 7,670 (10.49)
Degree		*n* = 70,735
Associate Bachelor of Science Master of Science Doctor of Physical Therapy EdD/PhD Other	33 (7.4) 76 (17.1) 74 (16.7) 207 (46.6) 43 (9.7) 11 (2.5)	2,949 (4.17) 16,466 (23.28) 12,005 (16.97) 36,106 (51.05) 2,509 (3.51) 700 (0.99)
Years in practice		*n* = 66,740
< =5 6–10 11–20 >20	47 (10.6) 74 (16.7) 100 (22.5) 223 (50.2)	29,417 (44.08) 9,806 (13.23) 13,670 (20.48) 21,199 (31.76)
**APTA member**		
Yes No No response	332 (74.8) 104 (23.4) 8 (1.8)	73,139 (100.0) – –
Practice setting (can select more than 1)		*n* = 63,938
Outpatient/Wellness Acute care hospital Assisted living Inpatient Rehab Skilled nursing facility Home health Academic program Other	205 (46.2) 62 (14.0) 67 (15.1) 30 (6.8) 154 (34.7) 88 (19.8) 54 (12.2) 35 (7.9)	38,237 (59.80) 6,131 (9.59) – 2,253 (3.52) 3,417 (5.34) 4,503 (7.04) 4,107 (6.42) 5,290 (8.27)
Employment status		*n* = 63,745
Full-time Part-time/per diem/other Other	364 (82.0) 79 (17.8) 1 (0.2)	54,448 (85.42) 6,255 (9.81) 3,042 (4.77)
**Percent of time in patient care**		
0–25% 30–50% 55–75% 80–100%	134 (30.2) 39 (8.8) 68 (15.3) 203 (45.7)	N/A
**% caseload 65+**		
0–25% 30–50% 55–75% 80–100% No response	35 (7.9) 40 (9.0) 90 (20.3) 268 (60.4) 11 (2.5)	N/A

* *Sample size varies based on 2019 national data from the American Physical Therapy Association. Data available upon request only from American Physical Therapy Association (APTA) [Internet]. Contact APTA Staff; 2021. Available from: https://www.apta.org/need-help-contact-apta/email-freshdesk*.

**Figure 1:**
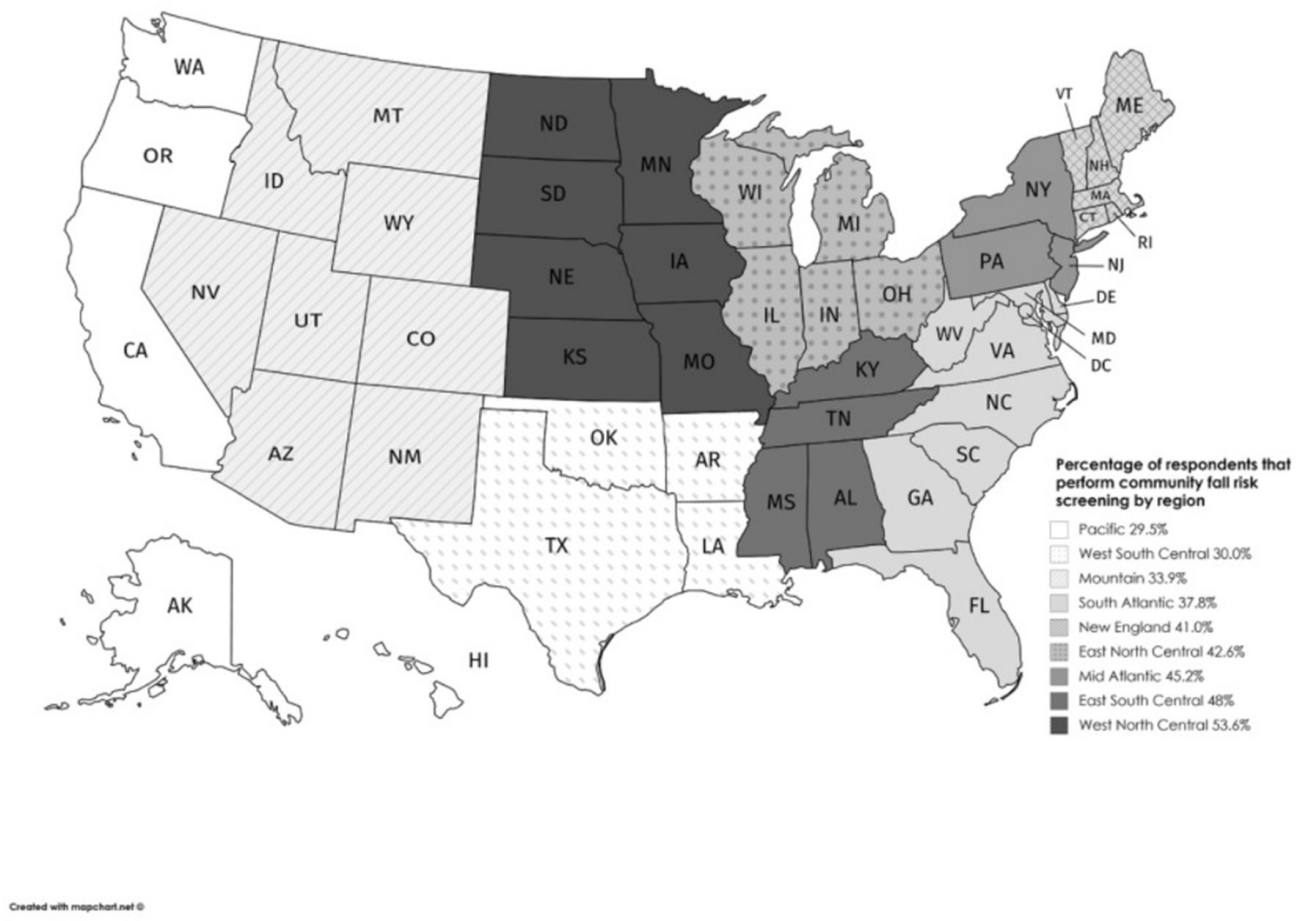
Percentage of physical therapists conducting community fall risk screenings by region.

Less than half of the respondents (*n* = 180, 40.5%) reported conducting community fall risk screenings. Only 14 of the 180 respondents who conducted falls screenings were PTAs (7.8%), which was 26.4% of the PTAs who responded to the survey (*n* = 53). The percentage of respondents who conducted falls screening by region is shown in Figure 1 (26). The Pacific region had the lowest percentage of therapists conducting community falls risk screenings (*n* = 13/44, 29.5%), whereas the West North Central region had the highest percent of respondents conducting screenings (*n* = 15/28, 53.6%). One survey respondent indicated they screened over one thousand older adults in the community each year.

Frequency of falls risk screening and knowledge of the STEADI tool of survey respondents who conduct falls risk screenings in the community are depicted in Table 2. Approximately 40% (*n* = 180) of survey respondents report conducting community falls risk screenings, either yearly (*n* = 96, 21.6%) or every 6 months (*n* = 49, 11.0%). Of the individuals who conduct screens (*n* = 180), most (*n* = 148) conduct <100 per year. The majority (*n* = 344, 77.5%) of survey respondents were somewhat to very familiar with the STEADI fall-risk screening toolkit and ~84% (*n* = 114) of respondents who were very familiar with the STEADI (*n* = 136) use it to conduct fall risk screenings. Therapists who conduct community-based falls risk screenings using the STEADI or some other screening tool (*n* = 125), report using the intervention/education components (*n* = 115, 92%) and three key screening questions (*n* = 97, 77.6%) of the STEADI most frequently.

**Table 2:** Frequency of Community Falls Risk Screening and Knowledge and Use of the STEADI Toolkit (*n* = 444).

**Survey item**	***n* (%)**
**Frequency of community fall risk screening**	
Do not screen	264 (59.5)
Screen	180 (40.5)
Monthly Every 6 months Yearly Other	15 (3.4) 49 (11.0) 96 (21.6) 20 (4.5)
**Number of individuals screened annually in community**	
Do not screen <25 26-100 >100	264 (59.5) 63 (14.2) 85 (19.1) 32 (7.2)
**How familiar are you with the STEADI as a tool for falls risk screening?**	
Very familiar Familiar Somewhat familiar Not familiar at all missing	136 (30.6) 87 (19.6) 121 (27.3) 99 (22.3) 1 (0.23)
**Do you use the STEADI tool for fall risk screening purposes?**	
**(Question only posed to respondents who indicated they were very familiar with STEADI**, ***n*** **=** **136)**	
Yes No Use other tool	114 (83.8) 11 (8.1) 11 (8.1)
**What components of the STEADI do you use? (could select more than 1 answer)** **(Question only posed to respondents who answered ‘Yes' or ‘Other' to using the STEADI**, ***n*** **=** **125)**	
Screening—stay independent brochure and questionnaire Screening-−3 key questions Functional mobility—Timed up and Go Functional mobility-−30-s chair stand test Functional mobility—four stage balance test Functional mobility—all three tests Assessment—multifactorial piece Intervention/Education (recommendations in algorithms/referrals & ways to decrease fall risk based on results)	62 (49.6) 97 (77.6) 84 67.2) 85 (68.0) 73 (58.4) 84 (67.2) 80 (64.0) 115 (92.0)

*Stopping elderly accidents, deaths, and injuries (STEADI).

Characteristics of respondents stratifying by whether or not they conduct falls risk screening in the community are presented in Table 3. Compared to individuals who do not conduct falls risk screening (*n* = 225), those who do (*n* = 166) are more likely to be a PT (*p* = 0.0256) and hold different terminal degrees than those who do not screen (*p* = 0.0400) with a slightly higher proportion with advanced academic degrees (MS/EdD/PhD). Likewise, those who conduct community fall risk screening reported different years in practice than those not (*p* = 0.0020) with a slightly higher proportion (*n* = 109, 60.6%) reporting >20 years in practice. Individuals who conduct community fall risk screening are also more likely to work in specific settings such as outpatient/wellness clinics or academic programs. Individuals who conduct screenings differed in the percentage of time spent in patient care (*n* = 77, 42.8%) than those not conducting screens (*n* = 57, 21.6%) with a slightly higher proportion reporting 25% or less of their time spent in patient care.

**Table 3:** Demographics of physical therapists and physical therapist assistants by community fall risk screening status.

	**Conduct Fall screening in community mean ± SD or *n* (%) (*n* = 180)**	**Does not Conduct fall screening in community Mean ± SD or n (%) (*n* = 264)**	***P*-value**
Age	48.4 ± 11.9	46.6 ± 11.8	0.1264
**Gender**			0.6298
Male Female Prefer not to report	26 (14.4) 152 (84.4) 2 (1.1)	47 (17.8) 214 (81.1) 3 (1.1)	
**Occupation**			**0.0256**
Physical therapist Physical therapist assistant	166 (92.2) 14 (7.8)	225 (85.2) 39 (14.8)	
**Degree**			**0.0400**
Associate BS MS DPT EdD/PhD Other	10 (5.6) 26 (14.4) 33 (18.3) 79 (43.9) 25 (13.9) 7 (3.9)	23 (8.7) 50 (18.9) 41 (15.5) 128 (48.5) 18 (6.8) 4 (1.5)	
**Years in practice**			**0.0020**
≤ 5 6–10 11–20 >20	12 (6.7) 28 (15.6) 31 (17.2) 109 (60.6)	35 (13.3) 46 (17.4) 69 (26.1) 114 (43.2)	
**APTA member**			0.0801
Yes No No response	137 (76.1) 37 (20.6) 6 (3.3)	195 (73.9) 67 (25.4) 2 (0.8)	
**Practice setting (can select more than 1)**			
Outpatient/Wellness	104 (57.8)	101 (38.3)	** <0.0001**
Acute care hospital	21 (11.7)	41 (15.5)	0.2488
Assisted living	36 (20.0)	31 (11.7)	**0.0170**
Inpatient Rehab	12 (6.7)	18 (6.8)	0.9502
Skilled nursing facility	51 (28.3)	103 (39.0)	**0.0202**
Home health	36 (20.0)	52 (19.7)	0.9373
Academic program	37 (20.6)	17 (6.4)	** <0.0001**
Other	15 (8.3)	20 (7.6)	0.7712
**Employment status**			0.1623
Full-time Part time/per diem/other No response	154 (85.6) 26 (14.4) 0 (0.0)	210 (79.6) 53 (20.1) 1 (0.4)	
**Percent of time in patient care**			** <0.0001**
0–25% 30–50% 55–75% 80–100%	77 (42.8) 18 (10.0) 26 (14.4) 59 (32.8)	57 (21.6) 21 (8.0) 42 (15.9) 144 (54.6)	
**% caseload 65+**			0.1125
0–25% 30–50% 55–75% 80–100% No response	11 (6.1) 13 (7.2) 33 (18.3) 121 (67.2) 2 (1.1)	24 (9.1) 27 (10.2) 57 (21.6) 147 (55.7) 9 (3.4)	

*American Physical Therapy Association (APTA) [Internet]. Explore APTA membership; 2021. Available from: www.apta.org/apta-and-you/explore-apta-membership/. Bolded values indicate statistical significance of p <0.05*.

As referral for appropriate interventions are important components after screening; in those survey respondents who report screening for falls in the community (*n* = 180) we examined their knowledge and use of community interventions (Table 4). The majority (*n* = 148, 82.2%) of respondents who report screening for falls risk in the community are aware that the National Council on Aging (NCOA) recommends EBPs for older adults and 75.6% (*n* = 136) refer to these programs. The most common referrals are to the Otago Exercise Program (*n* = 71, 39.4%), A Matter of Balance (*n* = 59, 32.8%), Tai Chi for Arthritis (*n* = 42, 23.3%) and YMCA Moving for Better Balance (*n* = 33, 18.3%). Individuals who screen for falls also refer to community programs and resources such as Silver Sneakers (*n* = 122, 67.8%) programming, senior centers (*n* = 116, 64.4%), local gyms (*n* = 103, 57.2%), and the YMCA (*n* = 75, 41.7%).

**Table 4:** Knowledge and use of community interventions among physical therapists and physical therapist assistants who conduct community-based fall screenings (*n* = 180).

	***n* (%)**
**Do you know the National Council on Aging recommends certain evidence-based health promotion/disease prevention programs for older adults?**	
Yes	148 (82.2)
No	32 (17.8)
**Do you ever refer to evidence-based health promotion/disease prevention programs?**	
Yes	136 (75.6)
No	30 (16.7)
I don't know	3 (17)
missing	11 (6.1)
**Which health promotion/disease prevention programs do you refer to in your community? (could select more than one)**	
A matter of balance	59 (32.8)^*^
Active choices	1 (0.6)
Active living every day	2 (1.1)
Arthritis foundation aquatic program	22 (12.2)
Bingocize	2 (1.1)^*^
CAPABLE (community aging in place—advancing better living for elders)	0^*^
Chronic disease self-management	14 (7.8)
Diabetes self-management	8 (4.4)
Enhance fitness	7 (3.9)^*^
Falls talk	4 (2.2)^*^
FallScape	0^*^
Fit and strong	10 (5.6)^*^
Geri-fit	6 (3.3)
Healthy moves for aging well	4 (2.2)
Healthy steps for older adults	6 (3.3)^*^
Healthy steps in motion	1 (0.6)^*^
On the move	4 (2.2)
Otago exercise program	71 (39.4)^*^
Stay active and independent for life	10 (5.6)^*^
Stepping on	22 (12.2)^*^
Tai chi for arthritis	42 (23.3)^*^
Tai Ji Quan: moving for better balance	21 (11.7)^*^
Walk with ease	8 (4.4)
YMCA moving for better balance	33 (18.3)^*^
Other	14 (7.8)
**What other community programs or resources do you typically refer your patients to? (could select more than one)**	
Local gym	103 (57.2)
Local senior center	116 (64.4)
Area agency on aging	52 (28.9)
YMCA	75 (41.7)
Silver sneakers	122 (67.8)
Other	23 (12.8)
I usually don't refer to community programs	11 (6.1)

* *Indicates this is an evidence-based program for fall prevention. YMCA (Young Men's Christian Association)*.

## Discussion

The aims of this study were to describe the characteristics of PTs and PTAs in the US who conduct community-based fall risk screenings, the reach of those screenings, and therapists' knowledge and utilization of the STEADI toolkit, evidence-based programs, and other community resources. We found that ~40% of PTs and PTAs who responded to this survey are conducting community fall risk screenings. Those who conduct screenings tend to be a PT, have over 20 years of experience, are involved in the least amount of direct patient care, and work in outpatient/wellness clinics or academic programs. The majority of PT/PTAs who do conduct community screenings do so once a year and screen between 26 and 100 older adults. Over 77% of respondents were somewhat to very familiar with the STEADI toolkit and over 80% of respondents who conduct community fall risk screenings and are very familiar with the STEADI use the toolkit. Of the PT/PTAs who conduct falls risk screenings in the community, over 75% are aware of and refer older adults to evidence-based health promotion/disease prevention programs and over half also refer to Silver Sneakers programming and/or local senior centers.

Considering that PTs in academia and who have the least direct patient-care hours are the most likely to conduct pro-bono community screenings, community organizations seeking PT partners to engage in community activities for older adults, such fall risk screenings, will likely have the most success collaborating with local physical therapy education programs or physical therapy clinic managers. Health science education often utilizes service learning to prepare students to practice in the healthcare environment. Many physical therapy programs in the United States have adopted service learning as a teaching and learning strategy, integrating real-life experiences to enrich the learning experience, teach civic responsibility, and strengthen communities (27, 28). Pro-bono community fall risk screenings and prevention activities are one approach to prepare students to function as members of an interprofessional healthcare team as well as how to engage with community organizations (28, 29). Physical therapy clinic managers in health systems and outpatient clinics engage in community screening events to support older adults and increase awareness of the services they provide.

The majority of PT/PTAs who conduct pro-bono community-based fall risk screenings in the United States engage in this service on an annual basis. The NCOA Falls Prevention Awareness Week initiative may account for most PTs/PTAs engaging in community-based fall risk screenings annually (30). The event occurs during the first week of fall and is heavily promoted by the APTA. PTs/PTAs collaborate with community organizations to provide annual falls prevention activities through multiple avenues; (1) local and/or regional Falls Free Coalitions (31), (2) State Physical Therapy Chapters (32), or (3) the American Physical Therapy Association-Geriatrics State Advocates (33). Physical therapists also may not be conducting fall screenings more frequently because they are required to achieve a high level of productivity in the clinic, and community screenings in the United States are pro bono services that do not generate revenue (34). Additionally, community screenings typically occur during the day, which is a barrier to PTs/PTAs participating since they are treating clinic patients and/or teaching physical therapy students during the daytime. Importantly, PTs value community engagement and proactive prevention and may be able to schedule screenings more than annually if approached by a community organization (35).

There was considerable geographic variability in the percentage of PTs/PTAs who conduct community-based fall risk screenings. For example, the Pacific region had the lowest percentage of respondents who conducted community falls risk screenings (29.5%), whereas the West North Central region had the highest percent of respondents who conducted screenings (53.6%). Besides the results being limited to the screening habits of only PTs/PTAs who responded to the survey, the reasons for geographic differences in screenings are unclear and likely multifactorial. States with wider public health initiatives around fall prevention may engage more PTs/PTAs in community screenings. Some state Fall Prevention Coalitions may include more PTs/PTAs and physical therapy academic programs as members, thereby increasing the likelihood that more community screenings are offered. State and/or regional health departments or Fall Prevention Coalitions may also have incorporated community screenings into their strategic plans and partnered with PTs/PTAs or academic programs to meet their objectives. State-supported universities with physical therapy education programs frequently have community engagement embedded in their missions (36, 37). Another factor that may contribute to the geographic variability in falls screenings may be differences in the outreach initiatives of various state chapters of the APTA. Further study of PTs/PTAs engagement in community screenings by state and regions should be conducted to better understand the facilitators and barriers to engagement. The geographic variability of our results should not prohibit community organizations from contacting potential PT/PTA partners in any region to engage in community-based fall risk screenings or other community events.

PTs are experts in movement, balance, strength, and exercise who can provide helpful strategies to prevent falls (18). Considering that over 75% of the all PTs/PTAs who responded in our study were somewhat to very familiar with the CDC-recommended STEADI toolkit and over 80% who were very familiar with the STEADI used the tool for screening, community organizations may engage PTs to provide fall prevention education to older adults and/or staff in addition to collaborating with PTs to conduct community fall risk screenings. PTs/PTAs may also serve as referral sources to EBPs considering that among therapists who report screening in the community, over 80% are aware that the NCOA recommends EBPs for older adults and 75% refer to these programs (19, 38, 39). The most common programs PTs/PTAs referred to were EBPs for fall prevention; the Otago Exercise Program, A Matter of Balance, Tai Chi for Arthritis, and YMCA Moving for Better Balance. Physical therapists likely reported a high referral rate to the Otago exercise program because they are one of the healthcare providers that can become certified to implement the one-on-one fall prevention program (40). Other programs that PTs commonly refer to, Matter of Balance and Tai Chi, are two of the most widely disseminated programs and therefore the most available to PTs/PTAs for referral. Besides referring to EBPs, survey respondents also refer to other community programs and resources such as Silver Sneakers, senior centers, local gyms, and the YMCA (41). These findings may be due to a number of reasons; first, survey respondents could indicate they referred to more than one program or site; therefore, they may be referring to multiple programs and resources. Second, YMCAs are in all 50 states and implement a number of EBPs (42). Silver Sneakers is available in many local senior centers and gyms and sometimes covered by Medicare Advantage Insurance (43); thus, these programs are accessible and affordable to many older adults. Finally, it is possible that some PTs are not aware of EBPs available in their area but are more aware of other local community resources such as YMCAs and Silver Sneakers. Outreach of community-based organizations to PTs/PTAs regarding available programs and resources may increase referrals and promote engagement of older adults.

### Strengths and Limitations

The strengths of this research include the contribution of new knowledge regarding the percentage and characteristics of PTs and PTAs in the United States who conduct pro-bono, community-based fall risk screenings and their knowledge and use of the STEADI and referrals to community resources. This information may help community organizations be more aware of PTs/PTAs' who are likely to engage and partner with for community screenings and EBP referrals. Additionally, the survey represents a large sample of PTs with respondents from 49/50 states. Limitations of this study include that, although there was representation from 49 states, the number of respondents varied from one to forty PTs in each state and a small number of PTAs. In addition, the low response rate and screening rate by PTAs lack generalizability. Survey respondents may not be representative of all PTs in the United States considering that the majority (75%) of our respondents were members of the APTA, whereas ~22% of all PTs in the United States are APTA members. Membership to the APTA provides PTs/PTAs with up to date clinical resources, education opportunities, and networking. Therapists must be members of the APTA to be members of their state physical therapy chapter as well. State chapters tend to facilitate engagement opportunities and events that are more regionally specific. State licensure is required for PTs and PTAs; however, membership to the APTA is voluntary and requires yearly renewal fees. There may be differences in community outreach among APTA members and non-members. Additionally, although our dissemination methods allowed for a broad reach, we were unable to determine how many PTs or PTAs received the survey and the response rate.

## Conclusion

The results of our study are the first to describe the characteristics of PTs/PTAs who conduct pro-bono community-based fall risk screenings, the number and geographic location of screenings, knowledge and use of the STEADI, and knowledge and referral practices to community resources. PTs and PTAs are key partners in evidence-based multifactorial fall prevention in the community. Data helps inform community organizations that most PTs performing community fall risk screening utilize the STEADI toolkit and refer to community-based programs. Community organizations seeking PT partners to engage in community fall risk screenings will likely have the most success collaborating with local physical therapy education programs or physical therapy clinic managers. Partnerships between community-based organizations and PTs can increase awareness and utilization of available community resources and have a positive impact on the health and well-being of older adults.

## Data Availability Statement

The original contributions presented in the study are included in the article/Supplementary Material, further inquiries can be directed to the corresponding author/s.

## Ethics Statement

Ethical review and approval was not required for the study on human participants in accordance with the local legislation and institutional requirements. Written informed consent for participation was not required for this study in accordance with the national legislation and the institutional requirements.

## Author Contributions

JV, TS, and JT: concept and design. SP, JB, JV, LS, and CH: analysis and interpretation of data. JV, JB, LS, and CH: manuscript preparation. All authors contributed to the article and approved the submitted version.

## Conflict of Interest

The authors declare that the research was conducted in the absence of any commercial or financial relationships that could be construed as a potential conflict of interest.
