# Supplementary figures and images for: Physical Therapists as Partners for Community Fall Risk Screenings and Referrals to Community Programs
Source: Front Public Health. 2021 Jun 25;9:672366. doi: 10.3389/fpubh.2021.672366 (PMC8267879; doi:10.3389/fpubh.2021.672366)

Supplemental File 1. CDC STEADI Algorithm for Survey


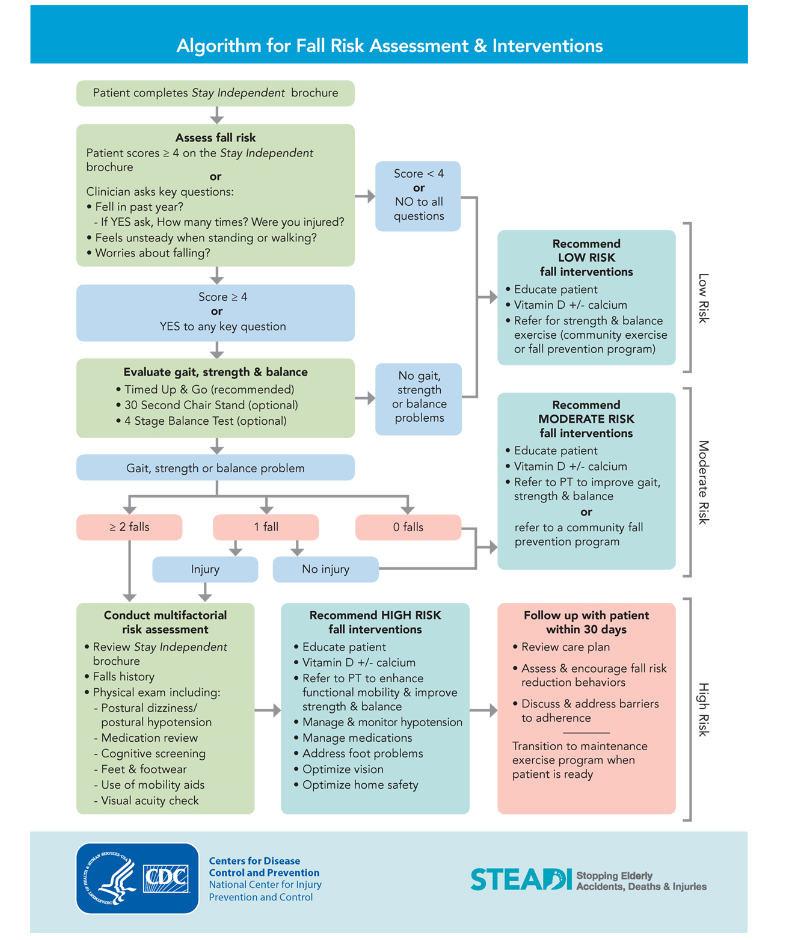

Supplement: Supplementary file 1 [file Table_1.DOCX]
